# Protocol for preparing fiber-defined simulated media for growth profiling of diverse human gut bacterial isolates

**DOI:** 10.1016/j.xpro.2026.104608

**Published:** 2026-05-29

**Authors:** Anjula Swathi Sagam, Gajender Aleti

**Affiliations:** 1 Department of Food and Animal Sciences, College of Agriculture, Tennessee State University, Nashville, TN 37209, USA

**Keywords:** Metabolism, Microbiology, Molecular Biology, Systems biology

## Abstract

Dietary fiber availability shapes gut microbial growth, yet standardized approaches for comparative growth profiling under defined fiber conditions remain limited. Here, we present a protocol for preparing fiber-defined simulated media based on yeast extract-casitone-fatty acid (YCFA) basal medium. We describe steps for anaerobic culturing of human gut bacterial isolates and inoculum normalization. We detail procedures for quantifying growth using optical measurements across contrasting fiber conditions. This scalable framework supports controlled investigation of diet-microbe interactions and microbial physiology.

## Highlights

• Standardized preparation of fiber-defined simulated media
• Controlled anaerobic culturing of diverse human gut bacterial isolates
• Reproducible OD_600_-based growth profiling across fiber conditions
• Adaptable for comparative microbial physiology studies

## Abstract (editor highlights)

Publisher’s note: Undertaking any experimental protocol requires adherence to local institutional guidelines for laboratory safety and ethics.

## Graphical abstract

**Figure:**
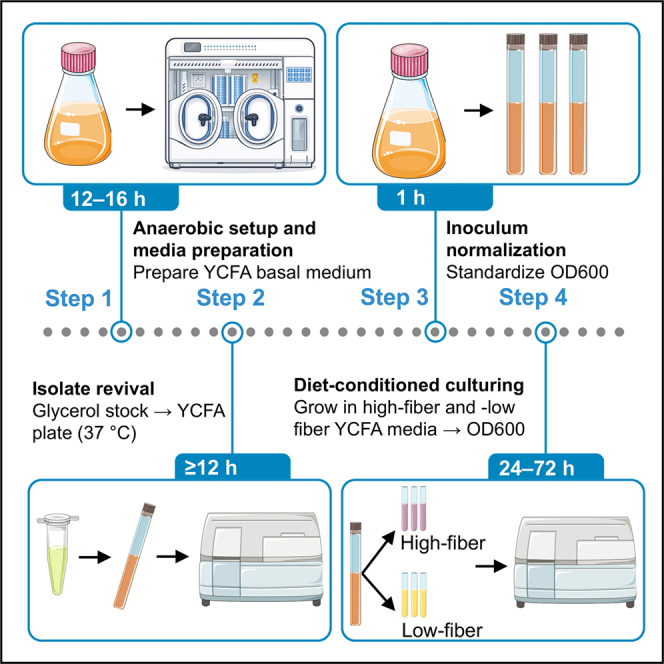

## Before you begin

The protocol below describes preparation of fiber-defined simulated media and comparative growth profiling of human gut bacterial isolates under controlled anaerobic conditions. The basal medium used throughout this protocol is Yeast extract–Casitone–Fatty acid (YCFA) medium, a widely used anaerobic formulation that supports robust growth of phylogenetically diverse gut isolates *in vitro*.

This workflow was developed to evaluate microbial growth dynamics under contrasting substrate environments that model differences in dietary fiber availability. While basal anaerobic media such as YCFA provide nutrients sufficient for general cultivation, they are not specifically configured for structured comparison of growth responses under defined fiber-enriched versus low-fiber conditions.

In this protocol, YCFA is adapted to generate fiber-defined simulated media that differ primarily in fiber complexity and carbohydrate composition while maintaining constant buffering capacity, mineral composition, vitamin supplementation, and reducing conditions. Standardized inoculum normalization controlled anaerobic handling, and media-specific optical density blank correction are implemented to ensure reproducible comparison across isolates and dietary conditions.

Although optimized for phylogenetically diverse anaerobic gut isolates, this protocol can be adapted to additional obligate or facultative anaerobic species with appropriate adjustment of inoculum density and incubation time.

### Benchmarking growth in YCFA basal medium

**Figure 1:**
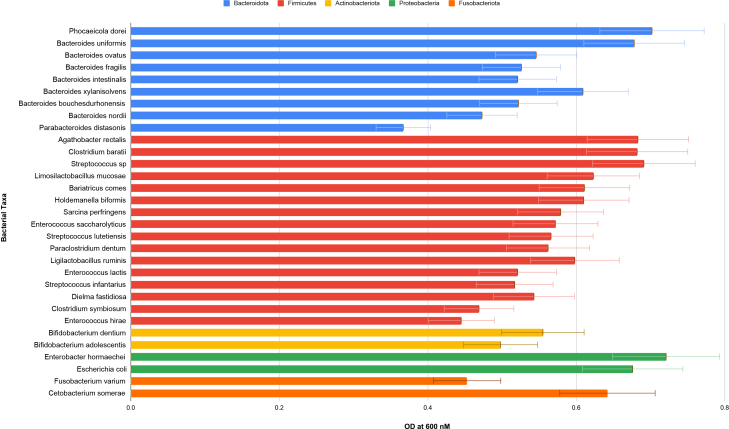
Growth of human gut bacterial isolates in YCFA basal medium Optical density (OD_600_) measurements at 24 h following anaerobic incubation in YCFA basal medium. Each bar represents the mean OD_600_ of three technical replicates for each isolate. YCFA supported robust growth across phylogenetically diverse human gut bacterial isolates, establishing it as a benchmark anaerobic cultivation system.

To contextualize the fiber-defined framework relative to a commonly used anaerobic cultivation system, we evaluated growth of the same human gut bacterial isolates in YCFA basal medium. YCFA supported robust growth across phylogenetically diverse isolates (Figure 1), consistent with its established use as a general-purpose anaerobic medium. However, YCFA is not explicitly configured to implement defined high-versus low-fiber contrast conditions. This benchmarking analysis establishes a growth-supportive baseline and motivates the fiber-defined contrast framework described below.

### Innovation

Basal anaerobic media such as Yeast extract–Casitone–Fatty acid (YCFA)is widely used to support *in vitro* cultivation of phylogenetically diverse human gut bacteria.^1^^,^^2^ In agreement, our benchmarking analysis showed that YCFA supported robust growth across diverse gut-derived isolates (Figure 1). However, YCFA lacks defined microbiota-accessible carbohydrate (MAC) substrates and therefore does not enable substrate-specific growth differentiation.

Although carbohydrate components are included in many basal formulations, they are typically combined with abundant peptones, yeast extract, and short-chain fatty acids that provide multiple overlapping carbon sources. As a result, systematic strain-level comparison under controlled fiber-enriched versus low-fiber conditions is not explicitly standardized within commonly used cultivation systems (Figure 1).

This protocol introduces a standardized, fiber-defined dietary contrast framework for cultivating human gut bacteria under controlled substrate conditions. While individual practices such as inoculum normalization, media-specific blank correction, and dietary contrasts are commonly used, they are not systematically integrated in existing workflows. Here, these elements are combined within a unified experimental design built upon YCFA, in which basal medium composition, buffering capacity, mineral content, and reducing conditions are held constant while fiber composition is selectively varied. This enables controlled comparison of microbial growth responses to defined microbiota-accessible carbohydrate (MAC) substrates. The protocol further incorporates standardized inoculum density and condition-specific background correction to improve reproducibility and quantitative comparability across isolates and experimental conditions. Together, this framework enables strain-resolved growth profiling under defined dietary contrasts. This approach is scalable and compatible with downstream metabolomic, genomic, and host-interaction assays, providing a reproducible platform for studying diet–microbiome interactions.

### Institutional permissions

This study does not involve human or animal subjects. All bacterial strains were obtained from publicly available repositories and handled in accordance with institutional biosafety guidelines at Tennessee State University.

Prior to beginning the protocol, complete the preparatory steps outlined below.

### Stabilization of the anaerobic workstation

**Timing:** 15 min to 12 h

This preparation establishes stable anaerobic conditions prior to media preparation and bacterial culture.

1. Initialize the anaerobic workstation.
  a. Verify proper function of the anaerobic workstation and confirm that oxygen levels are <1 ppm as indicated by the anaerobic sensor.
  b. Switch on the anaerobic workstation and confirm the premixed anaerobic gas cylinders are securely connected.
  c. Verify the gas mixture is set to 10% hydrogen, 10 % carbon dioxide and 80% nitrogen.
  d. Allow the internal atmosphere to stabilize until oxygen levels reach anaerobic conditions as indicated by the oxygen sensor.

**CRITICAL:** Ensure hydrogen concentration does not exceed 10%. Hydrogen is required for the catalytic oxygen removal but excessive may create safety risks.

***Note:*** Stabilization time varies depending on previous chamber usage and airlock activity.

***Note:*** The anaerobic workstation has an inbuilt incubator set to 37°C. For OD_600_ measurements, a small aliquot is transferred into an Eppendorf tube and briefly removed from the workstation for measurement, ensuring the main culture remains under anaerobic conditions throughout.

2. Pre-reduce consumables.
  a. Place sterile culture tubes, media bottles, pipette tips, and other consumables inside the workstation.
  b. Allow materials to remain under anaerobic conditions for ≥12 h prior to use when possible.

***Note:*** Pre-reduction minimizes oxygen exposure during media preparation and inoculation.

**Pause point:** Consumables may remain inside the workstation 12 h.

### Preparation of the YCFA basal anaerobic medium

**Timing:** ≥12 h pre-reduction

This preparation generates the basal formulation used for fiber-defined modifications.

3. Prepare basal medium.
  a. Dissolve all YCFA basal components listed in the Materials and Equipment section (Table 1) in deionized water.

**Table 1:** YCFA basal medium composition and preparation (1 L final volume)

Component	Final concentration	Amount (per 1 L)
Casitone	10.0 g/L	10.0 g
Yeast Extract	2.5 g/L	2.5 g
Glucose	5.0 g/L	5.0 g
K_2_HPO_4_	0.45 g/L	0.45 g
KH_2_PO_4_	0.45 g/L	0.45 g
NaCl	0.9 g/L	0.9 g
MgCl_2_·6H_2_O	0.0375 g/L	0.0375 g
CaCl_2_·2H_2_O	0.09 g/L	0.09 g
NaHCO_3_	4.0 g/L	4.0 g
L-Cysteine·HCl	1.0 g/L	1.0 g
Distilled Water	—	992.525 mL

**After autoclave (add aseptically)**		

Simplified VFA mix	—	6.25 mL
Hemin	10 μg/mL	125 μL (80mg/mL stock)
Vitamin K_1_	1 μg/mL	100 μL (10 mg/mL stock)
Vitamin Mix	—	1 mL
  b. Adjust pH to 6.8–7.0.
  c. Bring to the final volume.
  d. Autoclave at 121°C for 15–20 min.
  e. Cool to ∼40°C and aseptically add heat-labile components.

**CRITICAL:** Add heat-labile components only after autoclaving and cooling.

4. Pre-reduce basal medium.
  a. Transfer the sterilized medium into the anaerobic workstation.
  b. Loosen caps slightly to allow oxygen removal.
  c. Allow the medium to equilibrate anaerobically ≥12 h.

**Pause point:** Pre-reduced basal medium may remain inside the workstation 12 h.

### Preparation of YCFA agar plates

5. Prepare YCFA medium as described above but add 1.5% agar (w/v) before autoclaving.
  a. After autoclaving and cooling to ∼50°C, add heat-labile components aseptically.
  b. Pour plates inside a biosafety cabinet and allow it to solidify completely before transferring them to the anaerobic workstation.
  c. Allow plates to solidify and transfer into the anaerobic workstation for pre-reduction ≥12 h before use.

***Note:*** YCFA agar plates support colony formation consistent with the liquid medium environment used throughout the protocol.

### Preparation of fiber-defined simulated media

**Timing:** ≥12 h pre-reduction

This preparation generates contrasting fiber-enriched and low-fiber simulated media.

6. Prepare fiber-defined media formulations.
  a. Modify the basal medium with fiber or alternative carbohydrate components as described in the Materials and Equipment section.
  b. Mix thoroughly using a magnetic stir plate for 10–15 minutes to disperse fiber components. Some turbidity is expected and does not indicate a problem.
  c. Adjust pH if necessary.
  d. Autoclave and cool as described for basal medium.
  e. Add heat-labile components aseptically.

***Note:*** Fiber-containing media may remain partially turbid after sterilization.

7. Pre-reduce fiber-defined media.
  a. Transfer media into the anaerobic workstation.
  b. Allow to equilibrate anaerobically ≥12 h prior to inoculation.

**CRITICAL:** Maintain consistent fiber concentrations across experimental batches.

### Recovery and normalization of bacterial inoculum

**Timing:** 1–2 days (strain dependent)

This preparation ensures consistent starting inoculum density across isolates.

8. Recover bacterial isolates.
  a. Remove frozen glycerol stock from −80°C storage.
  b. Keep the glycerol stock on ice throughout. Using a sterile loop or pipette tip, scrape frozen material quickly without fully thawing the stock.
  c. Immediately return the stock to −80°C storage.
  d. Inoculate into 5 mL pre-reduced YCFA basal medium in a sterile culture tube and incubate anaerobically at 37°C until growth is observed.

**CRITICAL:** Avoid repeated freeze–thaw cycles.

**Tip:** Maintain ≥3–5 replicate cryovials per strain in −80°C storage to preserve viability across multiple access events and to mitigate the effects of partial thawing during airlock gas exchanges.

9. Standardize inoculum density.
  a. Measure OD_600_ of actively growing cultures.
  b. Dilute cultures in pre-reduced basal medium to a consistent starting OD (e.g., 0.05–0.1).
  c. Mix cultures gently by pipetting up and down to ensure a homogeneous suspension prior to OD_600_ measurement.

**CRITICAL:** Use identical starting OD for all isolates to ensure valid cross-condition comparison.

## Key resources table

**Table:** 

REAGENT or RESOURCE	SOURCE	IDENTIFIER
**Bacterial and virus strains**		

*Enterococcus hirae*	Global Microbiome Conservancy/ OpenBiome^3^	FK00382291-1
*Bifidobacterium dentium*	Global Microbiome Conservancy/ OpenBiome^3^	FK00382291-2
*Bacteroides fragilis*	Global Microbiome Conservancy/ OpenBiome^3^	FK00382291-3
*Limosilactobacillus mucosae*	Global Microbiome Conservancy/ OpenBiome^3^	FK00382291-4
*Bariatricus comes*	Global Microbiome Conservancy/ OpenBiome^3^	FK00382291-5
*Bacteroides intestinalis*	Global Microbiome Conservancy/ OpenBiome^3^	FK00382291-6
*Phocaeicola dorei*	Global Microbiome Conservancy/ OpenBiome^3^	FK00382291-7
*Bacteroides uniformis*	Global Microbiome Conservancy/ OpenBiome^3^	FK00382291-8
*Bacteroides ovatus*	Global Microbiome Conservancy/ OpenBiome^3^	FK00382291-9
*Fusobacterium varium*	Global Microbiome Conservancy/ OpenBiome^3^	FK00382291-10
*Sarcina perfringens*	Global Microbiome Conservancy/ OpenBiome^3^	FK00382291-11
*Clostridium baratii*	Global Microbiome Conservancy/ OpenBiome^3^	FK00382291-12
*Enterobacter hormaechei*	Global Microbiome Conservancy/ OpenBiome^3^	FK00382291-13
*Ligilactobacillus ruminis*	Global Microbiome Conservancy/ OpenBiome^3^	FK00382291-14
*Enterococcus saccharolyticus*	Global Microbiome Conservancy/ OpenBiome^3^	FK00382291-15
*Bifidobacterium adolescentis*	Global Microbiome Conservancy/ OpenBiome^3^	FK00382291-16
*Paraclostridium dentum*	Global Microbiome Conservancy/ OpenBiome^3^	FK00382291-17
*Dielma fastidiosa*	Global Microbiome Conservancy/ OpenBiome^3^	FK00382291-18
*Streptococcus infantarius*	Global Microbiome Conservancy/ OpenBiome^3^	FK00382291-19
*Agathobacter rectalis*	Global Microbiome Conservancy/ OpenBiome^3^	FK00382291-20
*Parabacteroides distasonis*	Global Microbiome Conservancy/ OpenBiome^3^	FK00382291-21
*Bacteroides xylanisolvens*	Global Microbiome Conservancy/ OpenBiome^3^	FK00382291-22
*Bacteroides bouchesdurhonensis*	Global Microbiome Conservancy/ OpenBiome^3^	FK00382291-23
*Holdemanella biformis*	Global Microbiome Conservancy/ OpenBiome^3^	FK00382291-24
*Streptococcus sp*	Global Microbiome Conservancy/ OpenBiome^3^	FK00382291-25
*Enterococcus lactis*	Global Microbiome Conservancy/ OpenBiome^3^	FK00382291-26
*Streptococcus lutetiensis*	Global Microbiome Conservancy/ OpenBiome^3^	FK00382291-27
*Escherichia coli*	Global Microbiome Conservancy/ OpenBiome^3^	FK00382291-28
*Bacteroides nordii*	Global Microbiome Conservancy/ OpenBiome^3^	FK00382291-29
*Clostridium symbiosum*	Global Microbiome Conservancy/ OpenBiome^3^	FK00382291-30
*Cetobacterium somerae*	Global Microbiome Conservancy/ OpenBiome^3^	FK00382291-31

**Chemicals, peptides, and recombinant proteins**		

Casitone (not available)	Thermo Fisher Scientific	225930
Yeast Extract	Difco	212750
Peptone	Difco	0118-17-0
Glucose	Acros organics	50-99-7
Fructose	Acros organics	161350025
Sucrose	Sigma	S0389
K_2_HPO_4_	Acros organics	205935000
KH_2_PO_4_	Thermo Fisher Scientific	P285
NaCl	Sigma	S3014
MgCl_2_·6H_2_O	Acros organics	7791-18-6
CaCl_2_·2H_2_O (not available)	Sigma	223506
NaHCO_3_	Sigma	S8875
L-Cysteine·HCl	Thermo Fisher Scientific	A10389-22
Vitamin Mix	Sigma	MBD0063
Vitamin K_1_ or K_2_	Thermo Scientific	L10575.06
VFA Mix	Sigma	CRM46975
Palmitic acid	Thermo Scientific	129702500
Tween 80	Fisher Scientific	BP338
Ethanol (70%)	Fisher bioreagents	BP8203
Hemin	Thermo Scientific	A11165.06
Inulin; average molecular weight ∼5 kDa	MP Biomedicals	198971
Pectin; citrus-derived polysaccharide, ≥74% galacturonic acid; methoxylation typically in the range of ∼55–60%	Sigma	P9135
Potato Starch; amylose and amylopectin, representing a semi-crystalline plant-derived polysaccharide	Thermo Scientific	419695000

**Other**		

A25 anaerobic workstation	Don Whitley Scientific	–
10% H_2_, 10% CO_2_, 80% N_2_ anaerobic gas mixture	Global Calibration Gases LLC	M068976-T
Autoclave	Fisher Scientific	STE2422005
Water bath	Precision	51221061
Refrigerator and freezer (−20 C)	Magic Chef	HMDR1000WE
Freezer (-80 C)	Thermo Fisher Scientific	U86-17D43
Magnetic stir plate	Thermo Scientific	SP131325Q
Analytic balance	Voyager	V02140
pH meter	Fisherbrand™ accumet™ AE150 Benchtop pH Meter	13-636-AE150
Micropipettes (1 - 1000 μL)	eppendorf 0.5–10 μL, eppendorf2 - 20 μL, eppendorf20 - 200 μL, eppendorf100 - 1000 μL	H12085M, G09194M, R41034L, R29022L
Plate reader	BioTek Synergy H1 Multimode Reader	8041000
Spectrophotometer	Thermo Scientific	14385400
Culture tubes	Fisherbrand	14-961-25
Pipette tips	Corning 1 - 200 μL, Corning 100 - 1000 μL	#4711 21-197-8J
Microcentrifuge tubes	Fisherbrand	05-408-129
Cryovials	Fisherbrand	1050026
Media bottles	Fisherbrand	FB-800-1000, FB-800-500, FB-800 -100
Syringe filters	TISCH scientific	SF14506
Disposable gloves	Fisherbrand	19-130-1597C
Syringes	BD Syringes	309646
Centrifuge tubes	Avantor - 50 mL, Avantor - 15 mL	21008 - 242, 21008 - 216

## Materials and equipment

### Anaerobic culturing considerations

Anaerobic cultivation is performed using a gloved anaerobic workstation (A25 Anaerobic Workstation, Don Whitley Scientific).

• Incubation temperature is maintained at 37°C unless otherwise specified.
• All media, consumables, and heat-labile reagents are placed inside the anaerobic workstation for ≥12 h prior to use to allow pre-reduction.
• Culture vessels include sterile anaerobic culture tubes with a 5–10 mL working volume.

### Optical density measurement

• Optical density is measured at 600 nm (OD_600_) using a benchtop spectrophotometer with compatible cuvettes.
• Media-specific blanks (basal medium, low-fiber medium, and high-fiber medium) are measured at each timepoint to correct for background turbidity.
• Blank-corrected OD values are used for downstream growth analysis.

### Medium composition and preparation

The diet stimulated medium used in this protocol was developed by modifying previously described basal gut microbial YCFA culture medium.^1^ Each was prepared in its respective diet formulation, resulting in the reproducible anaerobic culture of gut bacterial isolates in both media. Medium composition differs primarily in fiber complexity, sugar composition, protein load and lipid availability while maintaining constant buffering capacity, vitamin supplementation, mineral composition and anaerobic reducing conditions. Complete compositions and preparation details are provided in Tables 1, 2, and 3. All component concentrations are listed per liter (per L) final volume.^4^

**Table 2:** Fiber-defined simulated media (1 L final volume) High-fiber medium

Component	Final concentration	Amount
Yeast Extract	2.5 g/L	2.5 g
Inulin	10 g/L	10 g
Pectin	3 g/L	3 g
Potato Starch	10 g/L	10 g
K_2_HPO_4_	0.45 g/L	0.45 g
KH_2_PO_4_	0.45 g/L	0.45 g
NaCl	0.9 g/L	0.9 g
MgCl_2_·6H_2_O	0.0375 g/L	0.0375 g
CaCl_2_·2H_2_O	0.09 g/L	0.09 g
NaHCO_3_	4 g/L	4 g
L-Cysteine·HCl	1 g/L	1 g
Distilled Water	—	992.6 mL

**Low-fiber medium**		

Yeast Extract	2.5 g/L	2.5 g
Peptone	10 g/L	10 g
Glucose	10 g/L	10 g
Fructose	10 g/L	10 g
Sucrose	10 g/L	10 g
Tween 80	0.05% (v/v)	0.5 mL
K_2_HPO_4_	0.45 g/L	0.45 g
KH_2_PO_4_	0.45 g/L	0.45 g
NaCl	0.9 g/L	0.9 g
MgCl_2_·6H_2_O	0.0375 g/L	0.0375 g
CaCl_2_·2H_2_O	0.09 g/L	0.09 g
NaHCO_3_	4 g/L	4 g
L-Cysteine·HCl	1 g/L	1 g
Distilled Water	—	989.1 mL

**After autoclaving (both media)**		

Simplified VFA mix	—	6.25 mL
Hemin	5 μg/mL	62 μL (80 mg/mL stock)
Vitamin K_1_	1 μg/mL	100 μL (10 mg/mL stock)
Vitamin Mix	—	1 mL
Palmatic acid (low-fiber only)	0.3 mg/mL	3 mL (100 mg/mL stock)

Formulations were designed to generate defined high-fiber and low-fiber conditions. Component concentrations were optimized empirically to support reproducible microbial growth under these conditions.

**Table 3:** Stock solutions and storage conditions

Low fiber stimulated diet medium composition - 1 L			
Stock/ reagent	Conc	Preparation	Storage
Hemin	80 mg/ mL	Dissolve in NaOH (0.05–0.1 M), filter sterilize with 0.22 μm	Aliquot, −20°C, light-protected
Vitamin K1	10 mg/mL	Dissolve in 100% ethanol	Aliquot, −20°C, light-protected
Palmitic acid	100 mg/mL	Dissolve in 95–100% ethanol with warming (60–70°C). Vortex well. Keep warm while dispensing to prevent precipitation.	Aliquot, −20°C

All prepared liquid media (YCFA basal, high-fiber, and low-fiber) may be stored at 4°C under aerobic conditions for up to one week. Re-introduce into the anaerobic workstation and allow ≥2 h of re-equilibration before use. YCFA agar plates may be stored inside the anaerobic workstation for up to 2 weeks.

## Step-by-step method details

### Anaerobic setup and media preparation

**Timing:** 1.5–2 days

**Timing:** 0.5-day setup and ≥12 h pre-reduction (step 1)

**Timing:** ≥12 h pre-reduction (step 2)

**Timing:** ≥12 h pre-reduction (step 3)

**Timing:** ≥12 h pre-reduction (step 4)

Here, we describe steps for establishing anaerobic conditions and preparing media for cultivation of gut bacterial isolates under controlled fiber-defined conditions.

1. Prepare the anaerobic system.
  a. Prepare the anaerobic chamber using a premixed anaerobic gas (10% H_2_, 10% CO_2_, 80% N_2_) and allow oxygen levels to stabilize below 1 ppm before use.
  b. Set incubation temperature to 37°C.
  c. Place empty sterile bottles/tubes, pipette tips, and heat-labile reagents (vitamin stocks, hemin stock, vitamin K stock, lipid stock) in the chamber 12 h to pre-reduce.

***Note:*** Pre-reduction minimizes oxygen exposure during downstream media preparation and inoculation.

**CRITICAL:** Ensure that the anaerobic atmosphere is fully established before introducing any media or reagents.

**Pause point:** Pre-reduced materials can remain in the anaerobic chamber 12–16 h before proceeding to Step 2.

2. Prepare YCFA basal medium.
  a. Prepare YCFA basal medium according to the composition listed in Table 1. Stock solutions are described in Table 3.
  b. Add medium components listed in Table 1 to deionized water and mix until fully dissolved.
  c. Adjust pH to 6.8–7.0.
  d. Bring to 400 mL with deionized water.
  e. Autoclave at 121°C for 15–20 min.
  f. Cool to approximately 40°C.
  g. Add heat-labile components aseptically (filter-sterilized stocks): Vitamin mix (1 mL/L), vitamin K, hemin.
  h. Transfer medium into the anaerobic workstation and loosen caps to allow oxygen removal.

**CRITICAL:** Do not add heat-labile components before autoclaving, as this may lead to degradation.

**Pause point:** Pre-reduced base medium can be stored in the anaerobic chamber 12–16 h before use.

3. Prepare YCFA agar plates.
  a. Prepare YCFA medium as in Step 2 but add 1.5% agar (w/v) to the components before autoclaving.
  b. Adjust pH to 6.8–7.0.
  c. Bring to 400 mL with deionized water.
  d. Autoclave at 121°C for 15–20 min.
  e. Cool to approximately 40°C.
  f. Pour into sterile petri dishes inside or immediately adjacent to the anaerobic workstation.
  g. Allow plates to solidify and equilibrate anaerobically 12–16 h before use.

***Note:*** Plates should be pre-reduced for ≥12 h to ensure anaerobic conditions at the agar surface before streaking.

4. Prepare fiber-defined simulated media
  a. Prepare fiber-enriched and low-fiber formulations according to Table 2 using stock solutions described in Table 3.
  b. Mix thoroughly to disperse fibers (expect some turbidity).
  c. Adjust pH to 6.8–7.0.
  d. Autoclave at 121°C for 15 min.
  e. Cool to 40°C.
  f. Add heat-labile components aseptically (vitamin mix, vitamin K, hemin).
  g. Transfer to anaerobic chamber and allow it to pre-reduce 12–16 h.

***Note:*** Fiber-containing media may remain partially turbid after sterilization, which does not indicate contamination.

**Pause point:** Fiber-defined media may remain in the anaerobic workstation 12–16 h prior to inoculation.

### Isolate revival and inoculum standardization

**Timing:** 2–4 days

**Timing:** 1–2 days (strain dependent) (step 5)

**Timing:** 1–2 days (strain dependent) (step 6)

**Timing:** 30–40 min (step 7)

This section describes recovery of bacterial isolates and preparation of standardized inocula to ensure reproducible growth comparisons across conditions.

5. Preparation of bacterial inoculum.
  a. Remove frozen glycerol stock from −80°C storage.
  b. Using a sterile loop or pipette tip, streak frozen material onto a pre-reduced YCFA agar plate immediately upon removal from −80°C.
  c. Immediately return the stock to −80°C storage.
  d. Seal plate and incubate anaerobically at 37°C until single colonies are visible (typically 24–72 h, strain dependent).

**CRITICAL:** Avoid repeated freeze–thaw cycles, as this reduces viability of anaerobic isolates.

***Note:*** Colony morphology and size will vary across strains. Select well-isolated, representative single colonies for downstream inoculation.

6. Liquid culture and transfer to log phase.
  a. Pick a single well-isolated colony from the YCFA agar plate using a sterile loop.
  b. Inoculate into a pre-reduced YCFA liquid medium (5 mL) inside the anaerobic workstation.
  c. Incubate 12–16 h at 37°C under anaerobic conditions.
  d. Transfer the 12–16 h culture 1% (v/v) (50 μL into 5 mL) into a fresh pre-reduced YCFA medium.
  e. Incubate anaerobically at 37°C, monitoring growth periodically.
  f. Measure growth until log phase is reached (OD_600_ approximately 0.6).

**CRITICAL:** Transfer cultures at log phase (OD_600_ 0.6) to fiber-defined conditions. Using cultures at a consistent physiological state ensures reproducible growth kinetics across strains.

7. Normalize inoculum (standard starting OD).
  a. Measure OD_600_ of actively growing cultures.
  b. Dilute each culture in a pre-reduced basal medium to a common starting OD (e.g., 0.05–0.1, strain dependent).
  c. Mix gently by swirling.

**CRITICAL:** Use the same starting OD for all isolates to ensure comparability across dietary conditions.

***Note:*** For all experiments, inoculum of equivalent OD_600_ were used to account for differences in growth between isolates or between culture conditions. Cultures were diluted to an OD_600_ of approximately 0.05–0.1 (depending on strain growth characteristics) using pre-reduced base medium, and were gently mixed by inverted rotation to avoid introducing any oxygen into the culture prior to inoculation**.**

### Diet-conditioned culturing and growth profiling

**Timing:** 1–3 days

**Timing:** 10–20 min setup + 24–72 h (strain dependent) (step 8)

**Timing:** 5–10 min per time point (step 9)

Here, we describe inoculation of fiber-defined media and quantification of bacterial growth under contrasting dietary conditions.

8. Diet-conditioned culturing.
  a. Label culture vessels by strain, medium condition, replicate number, and planned timepoints.
  b. Dispense pre-reduced fiber-enriched and low-fiber media into culture tubes (5–10 mL working volume).
  c. Add normalized inoculum at a fixed proportion (e.g., 1–5% v/v) to achieve the same starting OD_600_ across all conditions.
  d. Set up three technical replicates per strain and medium conditions.
  e. Incubate anaerobically at 37°C.

**CRITICAL:** Maintain strict anaerobic conditions throughout incubation to prevent growth variability.

9. Growth measurement by optical density.
  a. Prepare media-only blanks for each medium formulation (basal, low-fiber, high-fiber).
  b. At each timepoint (e.g., 0 h, 24 h, 48 h), measure OD_600_ for all samples.
  c. Calculate blank-corrected values using: OD corrected = OD sample − OD blank
  d. Record strain, medium, replicate, timepoint, raw OD, blank OD, and corrected OD in a structured data sheet.

***Note:*** Technical replicates are averaged prior to downstream statistical analysis to reduce measurement variability.

## Expected outcomes

**Figure 2:**
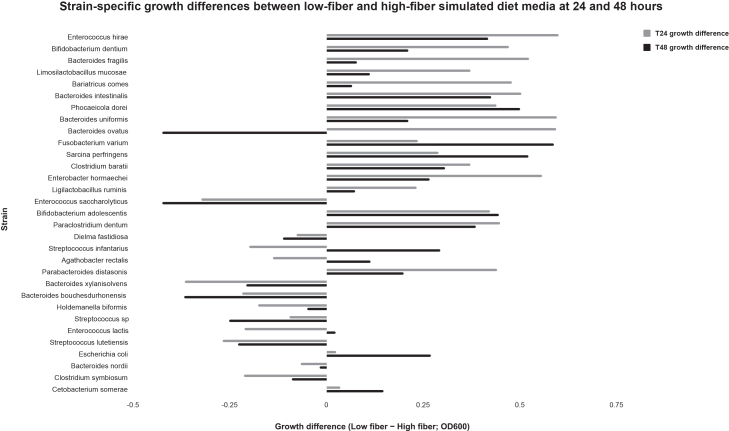
Strain-specific growth differences (ΔOD_600_; low-fiber minus high-fiber) at 24 and 48 h Positive values indicate enhanced growth under low-fiber conditions characterized by more readily bioavailable carbon sources, whereas negative values indicate enhanced growth under high-fiber, complex polysaccharide-rich conditions.

Using this protocol, human gut bacterial isolates are expected to grow reproducibly under anaerobic conditions in both YCFA basal and fiber-defined simulated media. Most strains exhibit visible turbidity within 12–48 h following inoculation, although growth kinetics vary depending on taxon and substrate composition. Differences in strain-level growth between low-fiber and high-fiber conditions can typically be observed at 24 and 48 h (Figure 2).

**Figure 3:**
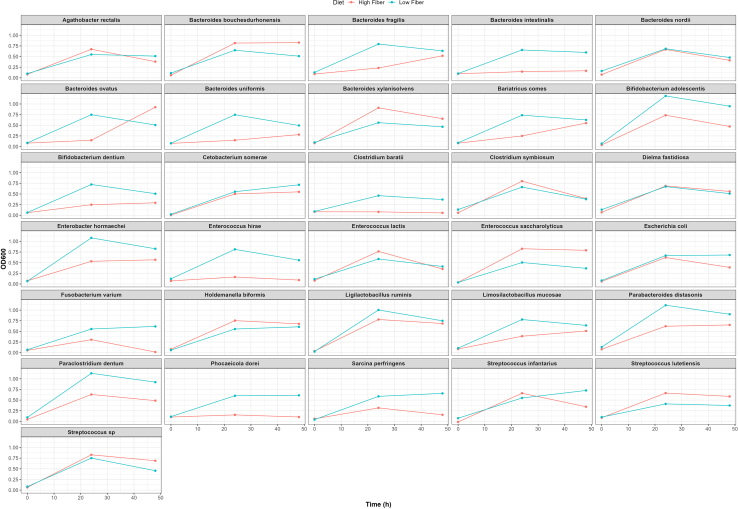
Diet-dependent growth dynamics of individual human gut bacterial isolates

Growth responses are quantified using OD_600_ measurements under anaerobic culture conditions. Blank-corrected OD values enable comparison of strain-specific growth dynamics across dietary substrate conditions. Differences are calculated as ΔOD_600_ (low-fiber minus high-fiber). Positive values indicate enhanced growth under low-fiber conditions characterized by more readily bioavailable carbon sources, whereas negative values indicate enhanced growth under high-fiber, complex polysaccharide-rich conditions. Representative strain-level growth curves across timepoints are shown in Figure 3.

Time-resolved OD_600_ measurements allow assessment of lag phase duration, growth rate, and final culture density. Biomass obtained from 5–10 mL anaerobic cultures is typically sufficient for downstream DNA or RNA extraction, depending on strain growth characteristics. When coupled with downstream molecular analyses, substrate-dependent differences in microbial growth may correspond to variation in gene expression or metabolic activity.

## Quantification and statistical analysis

Microbial growth was quantified using optical density measurement at OD 600.^5^^,^^6^^,^^7^ For each strain and dietary conditions, OD measurements were obtained in triplicates at each time point. Media specific blanks were measured and subtracted from raw OD values to correct for background turbidity. Blank corrected OD values were averaged across replicates prior to exporting and statistical analysis. Growth responses were calculated by subtracting OD values measured at 0 hours from measurements obtained at 24 and 48 hours. Because each bacterial isolate was evaluated under both high fiber and low fiber conditions, strain level growth responses were treated as paired biological observations. Growth differences between dietary media were evaluated using paired t tests. To account for potential non-normal distribution of growth responses, non-parametric Wilcoxon signed rank tests were additionally performed.

At 24 hours bacterial growth was significantly higher in Low fiber medium compared to high fiber medium (paired *t* test: t = 2.88, df = 30, p = 0.0073; Wilcoxon signed rank test: V = 386, p = 0.0070). Similarly at 48 hours, low fiber medium supported significantly greater bacterial growth (paired *t* test: t = 2.23, df = 30, p = 0.0336; Wilcoxon test: V =357, p = 0.0321). Technical replicates were averaged prior to statistical testing, and statistical significance was defined as p < 0.05. All statistical analyses were performed using R statistical software.

## Limitations

This protocol is based on the use of optical density measurements as a surrogate for bacterial growth, which may be affected by medium turbidity, especially in the presence of fibers. Although the use of blank subtraction helps to reduce such effects, OD600 is not able to differentiate between viable and non-viable cells and may not accurately reflect cell numbers for certain bacterial aggregates or biofilm-forming microorganisms. OD_600_ was selected to enable high-throughput comparison across multiple strains and conditions; CFU-based enumeration, while more direct, is less scalable under strict anaerobic workflows. Because technical replicates were averaged prior to analysis, reported p - values reflect strain level paired differences rather than replicate level variances.

The protocol is specifically designed for culturable anaerobic gut isolates, and it may not support the growth of fastidious or obligate syntrophic microorganisms without additional modifications to the medium. Moreover, it is possible that the use of fibers *in vitro* may not accurately predict microorganisms’ function in a complex *in vivo* environment, where both host and microbe may influence nutrient availability. Care must be taken to maintain anaerobic handling throughout the protocol, as microaerophilic or strict anaerobic microorganisms may be affected by even brief exposure to oxygen. In addition, it is worth noting that the rate of growth is strain-dependent, and incubation periods may vary when working with novel isolates. Across tested isolates, members of major gut-associated phyla, including Bacteroidota, Firmicutes, Actinobacteriota, Proteobacteria, and Fusobacteriota exhibited consistent growth under YCFA-based conditions; however, growth responses remain strain-dependent. Fiber components are derived from commercial sources and may exhibit batch-dependent variation in molecular weight and degree of substitution, which could influence microbial utilization.

## Troubleshooting

### Problem 1

Poor or no bacterial growth after inoculation.

### Potential solution

• Verify that media and inoculum were fully pre-reduced before inoculation.
• Confirm anaerobic chamber integrity and oxygen indicators.
• Extend incubation time for slow-growing strains.

### Problem 2

Large variability between replicates.

### Potential solution

• Reconfirm OD_600_ normalization accuracy before inoculation.
• Ensure equal inoculation volumes and thorough but gentle mixing.
• Avoid repeated opening of culture vessels during incubation.

### Problem 3

High background OD readings in fiber-defined media.

### Potential solution

• Prepare and measure media-only blanks for each fiber condition at every time point.
• Mix cultures gently before OD measurement to ensure uniform suspension.
• Interpret growth trends using corrected OD values rather than raw measurements.

### Problem 4

pH drift during incubation.

### Potential solution

• Confirm initial pH adjustment before autoclaving.
• Ensure buffering components were added at correct concentrations.
• Reduce incubation duration if excessive acidification is observed.

### Problem 5

Oxygen exposure during handling.

### Potential solution

• Minimize time outside the anaerobic chamber.
• Use pre-reduced consumables and media whenever possible.
• Avoid vigorous vortexing or pipetting that introduces air.

### Problem 6

Wilcoxon warning about ties.

### Potential solution

• Ties occur when some strains show identical growth differences across conditions.
• R will report an approximate p - value.
• This does not invalidate the test.

### Problem 7

No colony growth on YCFA agar plates.

### Potential solution

• Confirm plates were fully pre-reduced before streaking.
• Verify that frozen stocks are viable by checking for previous successful cultures.
• Increase incubation time, as some strict anaerobes require 72 h or longer for visible colonies.
• Ensure YCFA agar plates were prepared with correctly autoclaved and supplemented medium.

## Resource availability

### Lead contact

Further information and requests for resources and reagents should be directed to and will be fulfilled by the lead contact, Dr. Gajender Aleti (galeti@tnstate.edu).

### Technical contact

Technical questions on executing this protocol should be directed to and will be answered by the technical contact, Dr. Gajender Aleti (galeti@tnstate.edu).

### Materials availability

This study did not generate any new unique reagents.

### Data and code availability

This study generated OD_600_ growth-profiling datasets and derived growth metrics. Supporting tables are provided as an Excel file with raw and blank-corrected OD values and summary growth metrics.

## Acknowledgments

This work was supported by the 10.13039/100020664USDA Evans-Allen Program (grant nos. 222725 and 222768) and the 10.13039/100000049National Institute on Aging (NIA) grant P30-AG086403.

## Author contributions

Conceptualization, G.A.; formal analysis, A.S.S. and G.A.; investigation, A.S.S. and G.A.; writing, A.S.S. and G.A.; revising, A.S.S. and G.A.; funding acquisition, G.A.

## Declaration of interests

The authors declare no competing interests.

## Supplemental information

Data S1. Raw OD600 measurements for all strains across timepoints and conditions (Sheets S1A and S1B), related to Step 9

Data S2. Blank-corrected OD600 values used for growth analysis across fiber-defined conditions, related to Step 9

Data S3. Growth data and summary metrics including ΔOD600 and replicate averages for each strain (Sheets S3A–S3B), related to Step 9

Data S4. Statistical analysis outputs including paired t test and Wilcoxon test results, related to quantification and statistical analysis
